# Families’ experiences of central-line infection in children: a qualitative study

**DOI:** 10.1136/archdischild-2022-324186

**Published:** 2022-07-21

**Authors:** Carmen Soto, Mary Dixon-Woods, Carolyn Tarrant

**Affiliations:** 1 Paediatric Oncology, University College London Hospitals NHS Foundation Trust, London, UK; 2 THIS Institute, University of Cambridge, Cambridge, UK; 3 Health Sciences, University of Leicester, Leicester, UK

**Keywords:** child health, health services research, paediatrics, qualitative research

## Abstract

**Objective:**

Central venous access devices (CVADs), often known as central lines, are important for delivering medically complex care in children, and are increasingly used for children living at home. Central line-associated bloodstream infection (CLABSI) is a serious, life-threatening complication. Although the physical consequences of CLABSIs are well documented, families’ views and experiences of CLABSI are poorly understood.

**Design:**

Qualitative study using semistructured interviews with participants from 11 families of a child living at home with a CVAD.

**Participants:**

Parents of children aged 4–12 years living at home with a CVAD. Four fathers and nine mothers participated in interviews.

**Results:**

The risk of CLABSI is a constant fear for families of a child with a CVAD. Though avoiding infection is a key priority for families, it is not the only one: maintaining a sense of ‘normal life’ is another goal. Infection prevention and control require much work and expertise on the part of families, contributing significantly to families’ physical and emotional workload.

**Conclusions:**

Living with the risk of CLABSI poses additional burdens that impact on the physical and emotional well-being of families. Services to better support families to manage these burdens are needed.

## Abstract (precis)

In this qualitative study using semi-structured interviews of caregivers of children with central-venous access devices, families report that living with the risk of central line-associated bloodstream infections poses important burdens on families that impact their physical and emotional well-being.

**What is already known on this topic**

- Growing numbers of children with central venous access devices (CVADs) live in the family home. CVADS carry high risks of central line-associated bloodstream infection (CLABSI), yet accounts of families’ experiences of device care, managing infection risk and having CLABSI remain poorly understood.

**What this study adds**

- The risk of CLABSI adds a significant burden to families caring for a child with a CVAD, undermining efforts at normalisation.

**How this study might affect research, practice or policy**

- Practical, evidence-based approaches for support families whose child is at risk of CLABSI are needed. Policies to manage CLABSI should consider the importance of normalisation for families to support risk stratification and consider early discharge from the hospital when a CLABSI is suspected.

## Introduction

Central venous access devices (CVADs) are often used in the care of children with medically complex needs arising from a wide range of different conditions (including cancer, chronic respiratory illness and gastrological dysfunction).^1^ These devices—which include tunnelled lines with external catheters (‘central lines’) or implanted under the skin (portacaths)^2^—are used to deliver medication (including chemotherapy), nutrition and to sample blood. Notwithstanding their many benefits, CVADs pose a serious risk of infection to children, with up to 2.58 infections/1000 central-line days observed in the ambulatory setting.^3^ The consequences of central line-associated bloodstream infections (CLABSIs) can be serious: up to 15% of children with these infections require admission to intensive care units, and in some groups, CLABSI-associated mortality may be as high as 7%.^4–6^ In addition to the immediate clinical impact, CLABSIs contribute to longer-term disability and are a leading contributor to the burden of healthcare-associated infections.^7^

Although no intervention has been shown to completely eliminate the risk of CLABSI, there is good evidence that strict adherence to infection control practices in hospital settings can reduce the incidence of infection.^8^ However, new and largely underexplored challenges are posed by the increasing numbers of children with a wide range of complex medical conditions requiring the use of CVADs who live at home, where everyday care of the device is undertaken largely by family members.^9–12^ In domestic settings, families caring for their child take on tasks normally undertaken by skilled nurses, including dressings, flushing lines, giving medications and connecting parenteral nutrition.^10 11 13^

The experiences of family members of CLABSI are likely be distinctive, given their responsibilities for infection prevention. Understanding families’ experiences of caring for children in these circumstances is crucial to informing the design of services to better support families, yet their views and experiences of CLABSIs have remained underexplored.^9^ Studies of patients’ experiences of healthcare-acquired infections to date have tended to focus instead on adult patients’ experiences in hospital settings, where most care is provided by healthcare professionals. This work indicates that healthcare-acquired infections may have profound social and psychological impacts.^14^ In some cases, patients blame healthcare professionals for their infection, can feel unsafe and may lose trust in those caring for them.^15 16^ In this article, we present an analysis of the CLABSI-related experiences (both prevention control and having an infection) of parents who are caring for children with a CVAD at home.

## Methods

We conducted a qualitative study involving semistructured interviews with parents and children. Families were eligible for inclusion if their child was aged between 4 years and 12 years, had a CVAD inserted for at least 3 months and was living at home.

The recruitment strategy was designed with input from parent groups. Families of children with CVADs were recruited from four NHS trusts in England or via online patient groups and networks in the UK. In the case of NHS trusts, information about the study was shared with families if the clinician felt it was appropriate to do so, thus ensuring that that families were not approached at times of crisis. Patient groups and networks were asked to share information about the study on their web pages, social media and newsletters.

Participant information for adults and children provided details of study purpose, the researchers’ backgrounds and experience, and motivation for the study. Families were not asked to make a formal expression of interest: the choice was left with them to contact the research team if they wished to discuss the study further, and there was no direct contact with the research team unless potential participants themselves made contact. All those who contacted the research team were eligible for the study and no further screening took place. This recruitment strategy meant that it was not possible to calculate response rates (eg, numbers of families who declined to participate).

Topic guides were developed for interviews with parents, informed by existing literature on healthcare-associated infections and parent experiences of caring for children with long-term health conditions,^9 11–27^ and discussion with patient groups (online supplemental appendix 1). Written consent was obtained for parent interviews. Children were invited to take part in this study, but their interviews focused on living with a CVAD. Neither were they asked about their experiences of CLABSI nor did they choose to volunteer these experiences during the interview, so data from the child interviews are not reported here.

10.1136/archdischild-2022-324186.supp1 Supplementary data

Interviews were conducted face-to-face in families’ own homes. They were conducted with two parents where possible, although in practice, most interviews were with one parent. Interviews were carried out in the family home—children and other family members were often present intermittently—and interviews were frequently interrupted. Interviews lasted between 40 min and 2 hours; most interviews were an hour long. All interviews were conducted by CS, who was a trainee in paediatrics and a PhD candidate at the time of the study; she had prior experience and training in interviewing young children and families in a research context. CS had no ongoing clinical relationship with any of the participants.

Interviews were digitally recorded and transcribed. A modified grounded theory approach was used involving identifying key concepts which emerged from the data; applying codes to each section of transcribed data; organising these codes into a structured coding tree using NVivo software (online supplemental materials); and using ‘free writing’ to generate these codes into theories, informed by existing literature.^28–30^ Analysis was carried out by CS, alongside data collection, and was informed by regular discussions with CT and MD-W; theoretical saturation was reached before the final interview was conducted.

Participants were not contacted to correct transcripts or to check findings, since discussion with parent groups suggested that this would be an additional burden for families. The study was carried out as part of a PhD.

## Results

Data from four fathers and nine mothers across 11 families are presented here. Participants’ children lived with a range of medical conditions requiring the use of CVAD (including cancer, respiratory conditions and congenital syndromes affecting different organs). All families lived in England in a range of family structures (table 1). The mother acted as the primary carer for most of the children in the study. Of the 11 participating families, 5 had experienced confirmed CLABSI, and 2 children had developed sepsis as a result.

**Table 1:** Interview participants

Child	Child participation	Reason for device insertion	Age	Gender	Parent(s) interviewed	Family structure
C1	Interview	Cancer	4	Female	M1 and F1	Lived with two parents and siblings, support from wider family
C2	Interview	Cancer	8	Male	M2	Lived with two parents and siblings, some support from wider family
C3	Interview	Respiratory	4	Male	F3	Lived with two parents and siblings, support from wider family
C4	Interview	Cancer	7	Female	M4 and F4	Lived with two parents and siblings, limited support from wider family
C5	Interview	Cancer	4	Female	F5	Lived with father, shared care with mother
C6	Child refused	Cancer	11	Male	M6	Lived with two parents and siblings, limited support from wider family
C7	Child refused	Cardiology	7	Male	M7	Lived with mother and siblings, mother as sole carer
C8	Interview	Syndrome	9	Male	M8	Lived with mother and siblings, some support from wider family
C9	Interview	Syndrome	12	Female	M9	Lived with two parents and siblings
C10	Parents felt not appropriate	Syndrome	8	Female	M10	Lived with two parents and siblings, support from wider family
C11	Parents felt not appropriate	Neurological	9	Male	M11	Lived with mother as sole carer

F, father; M, mother.

### Living with the risk of infection: fear and uncertainty

Parents consistently described living with the pervasive fear that their child might acquire an infection and become seriously ill or die as a result. This fear was *in addition* to the worry that families already experienced linked to their child’s underlying condition and treatment (box 1, quotes 1.1 and 1.2). The fear of infection was ever-present, even when families had no personal experience of a CLABSI. The unpredictability of infection—CLABSIs could occur without warning—meant that families experienced stress, anxiety and loss of control (box 1, quote 1.3) and worried that they would need to rush their child to hospital with suspected infection (box 1, quote 1.4). Families who had already experienced a serious CLABSI in their own children or had seen other children develop one described these infections as deeply frightening (box 1, quote 1.5), especially when children deteriorated rapidly (eg, as a result of sepsis). Children were sometimes so ill that parents believed they might die and were left with traumatic recollections of these events persisting over many years. Other infections were less dramatic, not requiring intensive care or resuscitation, but were still miserable and frightening experiences for children and their families.

Box 1 **Living with the risk of infection**

1.1 ‘…as a parent it’s horrible, it’s, you know, to kind of, because you can’t control, and you’re not in control, and I think that is what it comes down to, that you’re not in control of what’s, what’s happening, who’s doing what, and nothing happens quick enough!’ (M9)

1.2 ‘I always worry. I will always worry until that thing is out’. (M2)

1.3 ‘It [suspected infection] never got easier to deal with, because there was always that worry that, you know, maybe this time she’ll go in and it will be something really serious and she might not come out’. (F5)

1.4 ‘Every temperature you’re going to hospital, because you have to, just in case’. (M8)

1.5 ‘I know a couple of kids who’ve died so it’s always there, […] … And whenever you feel settled […], you’ll hear of another child who got an infection, didn’t make it’. (M8)

F, father; M, mother.

Removing an infected central line was a significant procedure requiring surgery under general anaesthetic. Inserting a new central line (if that was possible) involved further surgery and another stay in the hospital. Replacing the central line became more technically challenging each time, and there were limits to how many central lines could be inserted during a child’s lifetime. Given that children were often dependent on devices for their survival, families were worried that any device removal could have long-term implications for their health.

### Guilt and responsibility

Families described providing the majority of everyday central-line care and infection prevention and control (IPC) for their children with a CVAD. Parents felt responsible for ensuring that the device was kept clean and used safely, often taking many additional precautions to ensure that care was performed to a high standard, but this weighed heavily on them. Those whose children had acquired a CLABSI while at home described an overwhelming sense that they were to blame for not having provided adequate care (box 2, quote 2.1). Those whose children had not had a CLABSI lived in the anticipation of feeling guilty if an infection occurred (box 2, quotes 2.2 and 2.3).

Box 2 **Guilt and responsibility**

2.1 ‘You do, you blame yourself, and you know, you just think did I do this?’ (M9)

2.2 ‘You are the one responsible for him catching an infection because you have not done it properly and then you have to go back into hospital and have it all sorted’. (M2)

2.3 ‘I could never live with myself if he got an infection, [and] it was after I had done all the dressing change’. (M8)

2.4 ‘If someone else does them (line cares) and then he gets an infection, you’re like well did they do it wrong?’ (M11)

F, father; M, mother.

Despite the burdens of looking after the device, some parents were reluctant to share the responsibility with others. In particular, they did not always trust healthcare workers to apply the same level of care over infection control that they took themselves to protect their child. For example, one mother described how she dispensed with most of her son’s care package as she did not feel confident that the care assistants who visited the home would protect him from infection: she decided to undertake all his central-line care herself, so that she was reassured that the care would be carried out correctly (box 2, quote 2.4).

### Disruption to normal life

Families attempted to maintain a normal life for their children (eg, by sending them to school or arranging family holidays). However, these attempts were shaped by the pervasive fear of CLABSIs, which overshadowed efforts to ensure a normal family life. Families felt they had to plan their lives to manage IPC procedures and to ensure they could attend hospital rapidly if their child became unwell. Children were reliant on family members to carry out tasks which they had previously performed independently, limiting their normal development (box 3, quote 3.1) One father explained how the family limited travel to ensure that they remained within reach of a hospital at all times (box 3, quote 3.2). Another family moved house to be closer to the hospital so they could more easily access care in the event of an infection or other emergency, but then found that there were no school spaces available near their new home. Infection, when it occurred, was hugely disruptive; children could not attend school or other activities (box 3, quote 3.3). Children already spent much of their time in the hospital because of their underlying health condition, so admissions for a device-related infection were especially unwelcome, for example, disrupting normal routines, requiring parents to take time from work or periods of unpaid leave (box 3, quotes 3.4 and 3.5), and causing cancellation of parties, holidays and other events at short notice. Siblings’ activities were also affected.

Box 3 **Disruption to normal life**

3.1 ‘I am having to do what I did when he was a toddler and go in and supervise everything. Obviously while he was in there, I would be around anyway, but it was up to him to wash himself in the shower and now I have to supervise and make sure it [CVAD] is not wet’. (M2)

3.2 ‘I wouldn’t have dared to leave the country… I would have wanted to be within 10 miles of the nearest hospital’. (F5)

3.3 ‘We tried to get her back [to school] sooner, but she, she kept ending up back and forth from hospital with a lot of infections’. (F5)

3.4 ‘When she goes into hospital I take unpaid leave, so the financial side…’. (M9)

3.5 ‘I would get a call and say, you know, [child)’s ill, come home, we need, we need to take her to the hospital and then I’d have to drop everything, that would be me disappeared from work for two or three days, minimum… So I got quite a few meetings with managers, quite a few disciplinary letters and stuff like that’. (F5)

F, father; M, mother.

## Discussion

This study of families caring for a child with a CVAD found both having a child who experienced a CLABSI and living with the threat of CLABSI had a significant impact on families’ lives, adding to the emotional burdens experienced by families and disrupting their attempts to maintain a normal life.

This study shows that managing the risk of central-line infection increases the treatment burden that families carry, both in the physical workload required to manage infection risk and in the emotional worries that accompany this risk. Caring for a child with medically complex needs in itself carries a significant treatment burden which is both physical and emotional.^31 32^ These burdens increase the work that families undertake, making it harder to carry out care safely and efficiently.^33^ In cases where trust in professional carers had been damaged, families were left carrying these emotional and physical burdens alone, a finding well described in the literature on patient safety.^27^ Despite the growing numbers of children living at home with a central line, the treatment burden associated with infection prevention and management in this population has been poorly explored. Recognising the treatment burden associated with efforts to manage risks of CLABSI is the first step in enabling these burdens to be addressed.

We found that fear of infections linked to devices exacerbates families’ worries that they are unable to care for their child effectively.^18 34^ When infections occur, parents experience guilt and self-blame^19 20 35^ and may suffer enduring trauma. These findings suggest that parents of children who develop CLABSI experience emotional turmoil similar to those of healthcare professionals following a medical error, so-called ‘second victims’.^36^ Even parents who had not seen a CLABSI in their child did not escape fear: they lived with the threat. The impacts on their everyday lives were profound; families experienced ongoing anxiety and anticipated guilt. One practical response may be for professionals not only to share parents’ commitment to the importance of IPC but also to emphasise that CLABSIs may occur through no one’s fault since no intervention has yet been shown to completely eliminate risk.

Another important finding of our study was the extent to which normalisation—where families emphasise and pursue aspects of everyday lives that are considered important to normal childhood^21–23^—was disrupted both by IPC practices *and* by the fear of an infection. Families attempted to live a normal family life but were frustrated in these attempts by the measures they had to take to reduce the risk of infection. These measures increased restrictions on everyday family life and further threatened the attempts at normality, which children and parents valued.^37^ On the other hand, experiencing a CLABSI itself posed a direct threat to normalisation, resulting in unpredictable hospital admissions and children missing out on everyday family life.^21 24^ Infections could also undermine children’s growing sense of self-autonomy, an important step in their developmental progression.^22 25^ Parents thus had to balance threats to normalisation from both the risk of CLABSI and the IPC measures intended to reduce this risk.

The burden of treatment associated with central-line care might be mitigated by a deeper understanding of the priorities of children and families. Exploring approaches to supporting parents to integrate IPC into their life in ways that enabled preservation of some normality would be of value, as would efforts to limit the impact of CLABSI on everyday life (eg, developing risk stratification tools which support early discharge from hospital^38^).

This study has several limitations. Participants were recruited from a variety of sources and were, to a certain extent, self-selecting. It may be that families who volunteered to take part in this research represent a subgroup that is particularly concerned about CLABSI. Though children were invited to take part in the interviews, they were neither asked to nor did they volunteer their experiences of CLABSI or their fears regarding infection. We did not include wider family members, such as siblings or grandparents. The experiences here can only be a partial reflection of the impact that CLABSI has on families. Future research should explore the experiences of children themselves, the wider family and broader community, with a particular focus on practical actions that can support families.

## Conclusions

Living with the risk of CLABSI has a substantial impact on families of children with CVADs living at home, beyond the purely medical consequences of their treatment. Infection prevention and managing the risk of CLABSIs can disrupt normalisation. Improvements in services are needed to support families and children.

## Data Availability

Data are available upon reasonable request. A full copy of the thesis is available from the Leicester Research Archive (Eynon Soto, Dawn Carmen (2017): Partnerships between families and professionals: Managing risks of infection in children with invasive devices. University of Leicester. Thesis; https://hdl.handle.net/2381/40904). Full transcripts are not publicly available as this would risk the anonymity of the participants.
