# Supplementary material for: Families’ experiences of central-line infection in children: a qualitative study
Source: Arch Dis Child. 2022 Jul 21;107(11):1038–42. doi: 10.1136/archdischild-2022-324186 (PMC9606494; doi:10.1136/archdischild-2022-324186)

1 Supplementary material

1.1 Parent Interview Topic Guide

Background
What experience do you have of looking after a child with a central line?

Daily Life
1. Take me through your normal day with the line, from when [your child] gets up
2. What are your responsibilities for looking after the line?
3. Who else looks after the line?
4. What about when [child's] not at home?
5. Do you get help from anyone else with looking after the line?

Challenges
1. What are the challenges of looking after a child with a central line at home?
2. What kind of training did you get about looking after the line?
3. What kind of information or support did you get?
4. What about before the line was put in – did you get a chance to ask questions?
5. Did you understand what it was going to be like for you and [child], having a line in?

Partnership
1. Who works with you to look after the line?
2. Do you get help from anyone else with looking after the line?
3. How does [child] help with looking after the line?

Risk Perception
1. Are line infections a worry for you?
   a. Do you know anyone who's had a line infection?
   b. Has [child] ever had a line infection?
2. What are your worries about the line getting infected?
   a. At home?
   b. Outside of home?
   c. When the line is accessed?
3. Where do you think most line infections come from?

Future Directions
1. What do you think would help families to look after a child with a central line?
2. What do you think would help reduce central line infections?

Soto C, *et al. Arch Dis Child* 2022; 107:1038–1042. doi: 10.1136/archdischild-2022-324186

1.2 Coding Tree

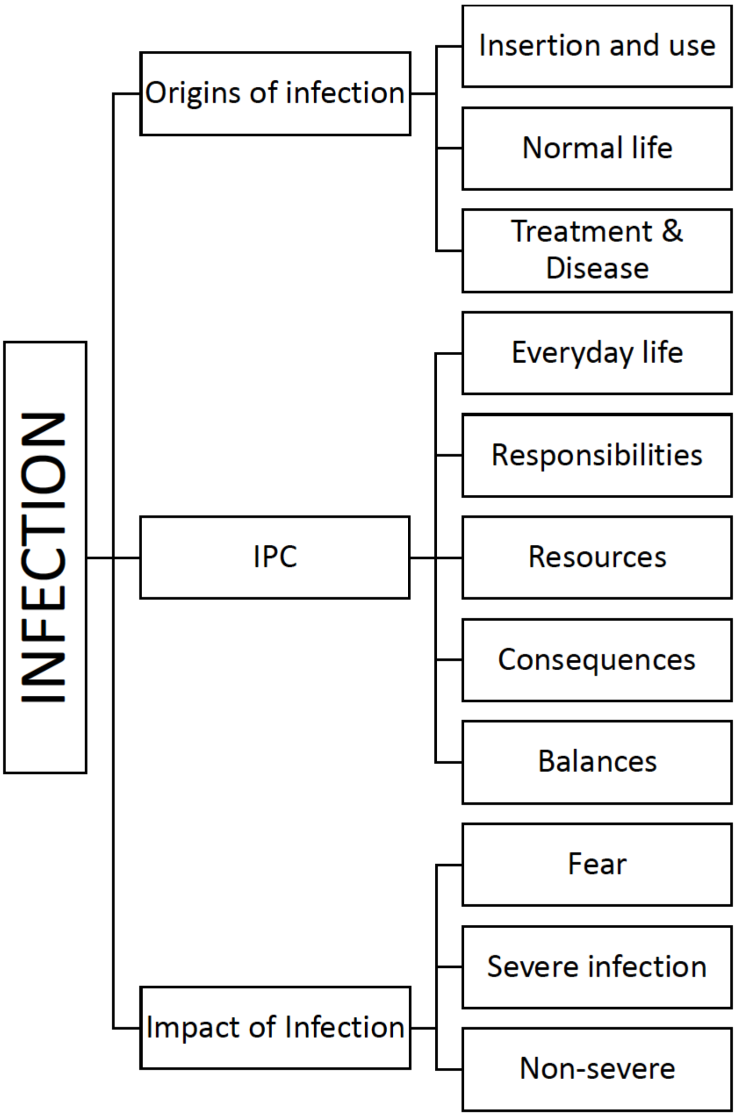

Supplement: Supplementary data [file archdischild-2022-324186supp001.pdf]
